# Positivity rates and subsequent patient dispositions after utilisation of cervical spine imaging referral guidelines in Singapore

**DOI:** 10.1186/s13244-025-02048-9

**Published:** 2025-08-08

**Authors:** Yi Xiang Tay, Shane J. Foley, Ronan Killeen, Marcus E. H. Ong, Robert Chun Chen, Lai Peng Chan, Eu Jin Tan, May San Mak, Wenlu Hou, Jonathan P. McNulty

**Affiliations:** 1 Radiography and Diagnostic Imaging, School of Medicine, University College Dublin, Dublin, Ireland; 2 Radiography Department, Allied Health Division, Singapore General Hospital, Singapore, Singapore; 3 St Vincent’s University Hospital, Dublin, Ireland; 4 School of Medicine, University College Dublin, Dublin, Ireland; 5 Department of Emergency Medicine, Division of Medicine, Singapore General Hospital, Singapore, Singapore; 6 Duke-NUS Graduate Medical School, Singapore, Singapore; 7 Department of Neuroradiology, Division of Radiological Sciences, Singapore General Hospital, Singapore, Singapore; 8 National Neuroscience Institute, Singapore, Singapore; 9 Department of Diagnostic Radiology, Division of Radiological Sciences, Singapore General Hospital, Singapore, Singapore

**Keywords:** Evidence-based practice, Clinical decision-making, Value-based health care, Radiology, Diagnostic imaging

## Abstract

**Objectives:**

This study seeks to evaluate the imaging characteristics and patient outcomes from the imaging recommendations of the ACR Appropriateness Criteria (AC), the ESR iGuide, and RCR iRefer.

**Materials and methods:**

This retrospective study evaluated cervical spine X-rays and CTs performed consecutively in a Singapore emergency department (ED) between October 1st and December 31st, 2022. Patient demographics, clinical diagnosis, ED clinical notes, and radiological findings were extracted from the electronic health record and subsequently reviewed to determine the associated imaging recommendations.

**Results:**

452 (mean age, 56 ± 17.3 years, 54.9% female) and 153 (mean age, 52.8 ± 21.4 years, 65.4% male) patients underwent X-ray and CT cervical spine, respectively. According to ACR AC and ESR iGuide, the positivity rate (4.3–7.2%) was the highest for appropriate studies and the lowest (0%) for inappropriate studies. For RCR iRefer, positivity rates (1.1–7.0%) were only observed for imaging classified as “*Indicated only in specific circumstances”*. There was a minimal difference in the proportion of patients with radiological findings that were categorised as positive and negative across the recommendations from the three guidelines. Most patients with inappropriate imaging in the X-ray cohort were discharged home or referred to specialists, whereas those in the CT cohort were primarily admitted to the hospital for conditions unrelated to the cervical spine.

**Conclusions:**

Inappropriate cervical spine imaging was associated with a lack of positive, significant findings. Imaging referral guidelines are specific and can effectively rule out significant pathology when imaging in the ED is not indicated. Clinical practice in the ED must incorporate imaging referral guidelines.

**Critical relevance statement:**

Imaging referral guidelines were effective in excluding a positive finding in traumatic and non-traumatic patients, especially when aligned with evidence-based clinical criteria.

**Key Points:**

- There are numerous imaging referral guidelines with unique methodologies, but the impact of individual imaging recommendations on imaging characteristics and patient dispositions remains unclear.
- There is minimal difference in the positivity rates across individual imaging recommendations from all three imaging referral guidelines.
- Inappropriate cervical spine imaging was associated with a lack of positive, significant findings.
- Guidelines are still effective in excluding significant pathology when imaging is not indicated

**Graphical Abstract:**

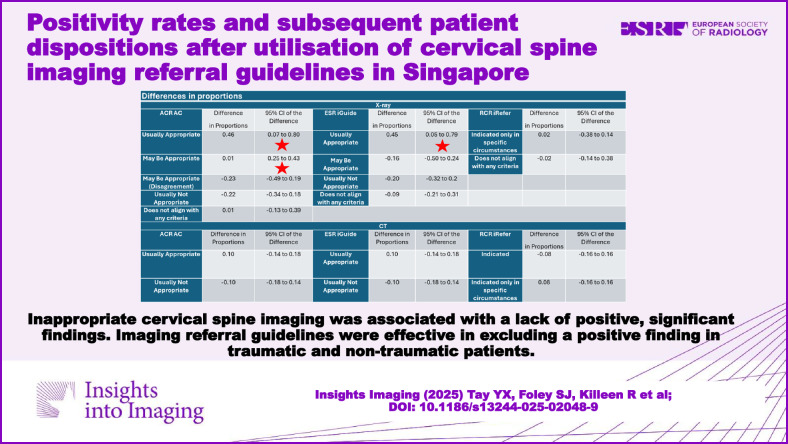

## Introduction

Diagnostic imaging in the emergency department (ED) is experiencing an upward trajectory in terms of utilisation [1]. Since 2010, about 50% of all ED visits in the United States have involved at least one imaging examination [2]. Imaging examinations can be categorised as either low-cost imaging, such as conventional radiography and sonography, or high-cost imaging, which encompasses computed tomography (CT) and magnetic resonance imaging [2]. The associated effects of ionising radiation, as well as the higher cumulative risk from examinations such as radiography and CT, are concerning [3].

Imaging referral guidelines empower clinicians to make appropriate imaging referrals by providing imaging recommendations for specific clinical scenarios. The imaging recommendations are derived from the most reliable evidence, with the incorporation of expert opinion and practice standards where applicable [4]. The American College of Radiology (ACR) Appropriateness Criteria (AC), the European Society of Radiology (ESR) iGuide, and the Royal College of Radiologists (RCR) iRefer are examples of imaging referral guidelines from prominent radiology professional bodies [5]. These guidelines can improve the appropriateness of imaging referrals and minimise unnecessary radiation exposure [6].

However, different imaging guidelines employed distinct methodologies for their content, resulting in varying imaging recommendations as observed in a recent publication by Tay et al [4, 7, 8]. The inclusion and/or exclusion of variables for considerations such as radiation risk, evidence, and cost awareness can directly affect the imaging recommendations. Furthermore, these imaging guidelines have variations in their imaging recommendations. This may lead to significant diversity in the emergency care provided and its associated outcomes, such as patient disposition (i.e. the destination of a patient in the care pathway following assessment and treatment in the ED) [9]. Consequently, this presents challenges in selecting, adapting, and adopting imaging referral guidelines for countries that have yet to adopt such evidence-based interventions, including Singapore.

We believe that imaging referral guidelines can effectively exclude significant positive findings in traumatic and non-traumatic patients, particularly in inappropriate ones. Accordingly, the primary outcome of the current study was to retrospectively examine the clinically significant positive and negative rates of cervical spine imaging (imaging characteristics) in the ED based on the imaging recommendations from ACR AC, ESR iGuide, and RCR iRefer. In tandem, we will also explore other outcomes, such as subsequent patient dispositions and imaging recommendations for the best imaging.

## Materials and methods

This retrospective clinical audit was approved by the SingHealth Centralised Institutional Review Board (CIRB) (No. 2020/2941). Based on this CIRB approval, a formal exemption from further full review/approval was granted by the University College Dublin Human Research Ethics Committee Sciences (HREC-LS) (Research Ethics Reference: LS-LR-23-82-Tay-McNulty).

As part of the audit, the study focused on the cervical spine, as it has been widely reported to have a high prevalence of low-value imaging [10]. We conducted a retrospective review of cervical spine X-rays and CT scans performed consecutively in a Singapore ED between October 1st and December 31st, 2022, regardless of patient presentation (traumatic and non-traumatic). For each imaging referral (Cervical Spine X-ray and CT Cervical Spine coded in the radiology information system), the initial clinical diagnosis (reasons for the referral), the ordered procedure, patient demographics (age and gender), ED clinical notes, and radiological reports were extracted from the electronic health records. Imaging referrals from general practitioners were excluded because we did not have access to the clinical notes for this specific group. No paediatrics were included in this review as the hospital does not provide medical care to patients under 12 years old.

Three authors (Y.X.T., S.F., J.M.) reviewed all imaging referrals, resolving disagreements through discussion and consensus. Based on the clinical diagnosis (reasons for the referral), the ordered procedure, patient demographics (age), and ED clinical notes, each imaging referral was matched with guidelines in ACR AC, ESR iGuide, and RCR iRefer, to assess whether the imaging was performed according to the imaging recommendation(s).

We also examined all imaging referral-associated radiological reports for significant positive findings (findings that will impact therapeutic decisions) as illustrated by Hoffman et al [11]. All radiological findings indicated in the radiological reports were also extracted. We recorded the information in a Microsoft Excel spreadsheet (Microsoft Corporation) for subsequent analysis. Y.X.T. reviewed the radiological reports and was blinded to the prior radiological report’s classifications.

We performed a descriptive analysis of the data, summarising categorical variables using frequency (percentages) and continuous variables using the mean (standard deviation). We also calculated the difference in proportion of the positive and negative radiological findings across the recommendations from the three imaging referral guidelines, i.e., ACR AC, ESR iGuide, and RCR iRefer. Since the ED had an attendance rate of 109,258 for the fiscal year concluding on March 31, 2022. Accordingly, 383 or more samples are needed to have a confidence level of 95%, that is, within a 5% margin of error to calculate the difference in proportion, as calculated in OpenEpi [12]. There was no missing data on the variables, as all required fields for analysis are available for extraction. All statistical analyses were performed in SPSS Statistics version 29.0 (IBM SPSS Inc).

## Results

### Study population

During the 3-month study period, 452 cervical spine X-rays and 153 CT cervical spines were performed within the ED, averaging six imaging referrals a day. The age range of patients included in this analysis was 17 to 99 years. The mean age of patients who underwent cervical spine X-ray was 56.0 (17.3 SD) years and 52.8 (21.4 SD) years for CT cervical spine, with a slight female preponderance (*n* = 248/452; 54.9%) for cervical spine X-ray and a male preponderance (*n* = 100/153; 65.4%) for CT cervical spine.

All patients who presented for a CT cervical spine were trauma patients, whereas the majority of patients (~ 70%) who presented for an X-ray had a variety of non-traumatic indications, such as radiculopathy. Electronic Supplemental Material Table 1 illustrates the indications for an X-ray and CT of the cervical spine in accordance with the three imaging referral guidelines.

### Cervical spine imaging characteristics, including positivity and negativity rates

64 (14.2%) and 70 (15.5%) X-ray referrals had imaging recommendations of *“Usually Appropriate”* based on ACR AC and ESR iGuide, respectively, while RCR iRefer did not have this category of recommendation. Likewise, based on ACR AC and ESR iGuide, there were 100 (22.1%) and 91 (20.1%) cases that were *“Usually Not Appropriate*”. According to RCR iRefer, the majority of the cases (*n* = 444/452; 98.2%) had the recommendation of *“Indicated only in specific circumstances”*. Only a small percentage of cases (1.3–9.1%) did not meet any of the criteria.

139 (90.8%) CT referrals had imaging recommendations of *“Usually Appropriate”* according to ACR AC and ESR iGuide, while RCR iRefer did not have this category of recommendation. Based on RCR iRefer, 11 (7.2%) and 142 (92.8%) cases had recommendations *“Indicated”* and *“Indicated under specific circumstances”*, respectively. A small number of cases (*n* = 14/153; 9.2%) were classified as *“Usually Not Appropriate”* by ACR AC and ESR iGuide. Tables 1 and 2 illustrate the imaging characteristics based on imaging recommendations.

**Table 1:** Imaging characteristics based on imaging recommendation(s)—ACR Appropriateness Criteria and ESR iGuide

X-ray							
**ACR AC**	***n*** ***(n/452, %)***	**+ve**^**a**^	**−ve**^**b**^	**ESR iGuide**	***n*** ***(n/452, %)***	**+ve**^**a**^	**−ve**^**b**^
Usually appropriate	64 (14.2%)	*n* = 3/64; 4.7%	*n* = 61/64; 95.3%	Usually appropriate	70 (15.5%)	*n* = 3/70; 4.3%	*n* = 67/70; 95.7%
May be appropriate	88 (19.5%)	*n* = 1/88; 1.1%	*n* = 87/88; 98.9%	May be appropriate	250 (55.3%)	*n* = 2/250; 0.8%	*n* = 248/250; 99.2%
May be appropriate (disagreement)	194 (42.9%)	*n* = 1/194; 0.5%	*n* = 193/194; 99.5%				
Usually not appropriate	100 (22.1%)	*n* = 0/100; 0%	*n* = 100/100; 100%	Usually not appropriate	91 (20.1%)	*n* = 0/91; 0%	*n* = 91/91; 100%
Does not align with any criteria	6 (1.3%)	*n* = 0/6; 0%	*n* = 6/6; 100%	Does not align with any criteria	41 (9.1%)	*n* = 0/41; 0%	*n* = 41/41; 100%

CT							
**ACR AC**	***n*** ***(n/153, %)***	**+ve**^**a**^	**−ve**^**b**^	**ESR iGuide**	***n*** ***(n/153, %)***	**+ve**^**a**^	**−ve**^**b**^
Usually appropriate	139 (90.8%)	*n* = 10/139; 7.2%	*n* = 129/139; 92.8%	Usually appropriate	139 (90.8%)	*n* = 10/139; 7.2%	*n* = 129/139; 92.8%
Usually not appropriate	14 (9.2%)	*n* = 0/14; 0%	*n* = 14/14; 100%	Usually not appropriate	14 (9.2%)	*n* = 0/14; 0%	*n* = 14/14; 100%

^a^ Radiological findings of fracture and soft tissue swelling

^b^ All other radiological findings

**Table 2:** Imaging characteristics based on imaging recommendation(s)—RCR iRefer

X-ray			
**RCR iRefer**	***n*** ***(n/452, %)***	**+ve**^**a**^	**−ve**^**b**^
Indicated only in specific circumstances	443 (98.0%)	*n* = 5/443; 1.1%	*n* = 438/443; 98.9%
Does not align with any criteria	9 (2.0%)	*n* = 0/9; 0%	*n* = 9/9; 100%

CT			
**RCR iRefer**	***n*** ***(n/153, %)***	**+ve**^**a**^	**−ve**^**b**^
Indicated	11 (7.2%)	*n* = 0/11; 0%	*n* = 11/11; 100%
Indicated only in specific circumstances	142 (92.8%)	*n* = 10/142; 7.0%	*n* = 132/142; 93.0%

^a^ Radiological findings of fracture and soft tissue swelling

^b^ All other radiological findings

For X-ray cervical spine using the ACR AC and ESR iGuide, the positivity rate was highest (ACR AC: 4.7% and ESR iGuide: 4.3%) in the category of *“Usually Appropriate”*, while the lowest positivity rate (0%) was in the category of *“Usually Not Appropriate”*. With respect to the RCR iRefer, the positivity rate was 1.1% for the only category of *“Indicated only in specific circumstances”*.

Notably, we observed a 0.45 (95% CI, 0.05 to 0.79) and 0.46 (95% CI, 0.07 to 0.80) difference in the proportion of radiological findings categorised as positive (ESR: *n* = 3/70, ACR: *n* = 3/64) and negative (ESR: *n* = 67/70, ACR: *n* = 61/64), respectively, of which the procedures were classified by the guidelines as “*Usually Appropriate*”. Additionally, there was a 0.02 (95% CI, −0.38 to 0.14) difference in proportion for procedures classified according to RCR iRefer as “*Indicated only in specific circumstances*”.

In terms of the CT cohort, the positivity rate was the highest in the category of *“Usually Appropriate”* in both ACR AC (7.2%) and ESR iGuide (7.2%). The *“Indicated only in specific circumstances”* category for RCR iRefer had the highest positivity rate (7%), and the category *“Indicated”* showed no positive findings.

In particular, we observed a 0.1 (95% CI, −0.14 to 0.18) difference in the proportion that were categorised as positive (ACR: *n* = 10/139, ESR: *n* = 10/139) and negative (ACR: *n* = 129/139, ESR: *n* = 129/139), respectively, that were classified as “*Usually Appropriate*”. Conversely, there was a −0.08 (95% CI, −0.16 to 0.16) difference in proportion for “*Indicated*” procedures classified according to RCR iRefer. A −0.1 (95% CI, −0.18 to 0.14) difference in proportion was also observed for inappropriate CT cervical spine classified as “*Usually Not Appropriate*”.

Table 3 illustrates the estimated difference in proportions between positive and negative radiological findings and the associated 95% confidence interval.

**Table 3:** Differences in proportions between positive and negative radiological findings

ACR AC	Difference in proportions	95% CI of the difference	ESR iGuide	Difference in proportions	95% CI of the difference	RCR iRefer	Difference in proportions	95% CI of the difference
X-ray								
Usually appropriate	0.46	0.07 to 0.80^a^	Usually appropriate	0.45	0.05 to 0.79^a^	Indicated only in specific circumstances	0.02	−0.38 to 0.14
May be appropriate	0.01	0.25 to 0.43^a^	May be appropriate	−0.16	−0.50 to 0.24	Does not align with any criteria	−0.02	−0.14 to 0.38
May be appropriate (disagreement)	−0.23	−0.49 to 0.19	Usually not appropriate	−0.20	−0.32 to 0.2			
Usually not appropriate	−0.22	−0.34 to 0.18	Does not align with any criteria	−0.09	−0.21 to 0.31			
Does not align with any criteria	0.01	−0.13 to 0.39						
CT								
Usually appropriate	0.10	−0.14 to 0.18	Usually appropriate	0.10	−0.14 to 0.18	Indicated	−0.08	−0.16 to 0.16
Usually not appropriate	−0.10	−0.18 to 0.14	Usually Not appropriate	−0.10	−0.18 to 0.14	Indicated only in specific circumstances	0.08	−0.16 to 0.16

^a^ Statistically significant finding

Five (1.1%) X-ray cervical spine and 10 (6.5%) CT cervical spine cases had positive significant findings (i.e., fracture or prevertebral soft tissue swelling were considered positive; all else considered negative). Fracture of the cervical spine was detected on two (0.4%) traumatic X-ray cervical spine and 10 (6.5%) traumatic CT cervical spines. Three (0.7%) non-traumatic X-rays of the cervical spine showed prevertebral swelling.

The most common non-significant findings in X-ray cervical spine were cervical spondylosis (84.5%), followed by loss of the normal cervical lordosis (33.2%) and spondylolisthesis (31.9%). For the CT cervical spine cohort, the most prevalent non-significant findings were cervical spondylosis (73.9%), followed by spondylolisthesis (26.1%). Ossification of the posterior ligament was depicted in X-ray and CT cervical spine, with a larger portion observed in the CT cohort (*n* = 19/153; 12.4%) than the X-ray cohort (*n* = 3/452; 0.7%). Electronic Supplemental Material Table 2 summarises the imaging characteristics for X-ray and CT cervical spine.

The majority of patients in the X-ray cervical spine cohort were either discharged home after treatment (ACR AC: *n* = 105/446; 23.5%, ESR iGuide: *n* = 103/411; 25.0%, and RCR iRefer: *n* = 103/443; 23.2%) or referred to specialists (ACR AC: *n* = 180/446; 40.4%, ESR iGuide: *n* = 160/452; 38.9%, and RCR iRefer: *n* = 179/443; 40.4%) for further evaluation. A subset of patients were either admitted to orthopaedics or other medical specialties for subsequent medical care. Only a small proportion of the patients were admitted to the observation ward in the ED for protocolised care (emergency observational medicine). In the X-ray cohort, the majority of patients with inappropriate imaging (or indicated only in specific circumstances) received home discharges or referrals to specialists for outpatient appointments.

Most of the patients in the CT cervical spine cohort were admitted to either orthopaedics or other medical specialties for further medical care. The remaining patients were treated and discharged, referred to specialists for further assessment, or admitted to the observation ward of the ED. Similar to the X-ray cervical spine group, there were also a few patients who wished to be discharged from the hospital despite the advice of the clinicians (Discharged, At Own Risk). Most patients in the CT cohort with inappropriate imaging (or indicated only in specific circumstances) had hospital admissions for conditions unrelated to the cervical spine. Electronic Supplemental Material Table 3 illustrates the patient disposition based on imaging recommendations.

The respective imaging referral guidelines recommended a test as a better imaging alternative to a cervical spine X-ray in 78.9% (ACR AC), 60.8% (ESR iGuide), and 80.6% (RCR iRefer) of cases. The number of cases in which the best imaging tests were available to replace the CT cervical spine was smaller, at 0.7% and 2%, as recommended by ESR iGuide and RCR iRefer, respectively. Table 4 summarises the best imaging tests available for replacement.

**Table 4:** Best imaging examinations according to imaging referral guidelines.

**Imaging recommendations for the best imaging tests for replacing X-ray Cervical Spine**					
**ACR AC**	***n*** **(*n*/446, %)**	**ESR iGuide**	***n*** **(*n*/411, %)**	**RCR iRefer**	***n*** **(*n*/443, %)**
MRI cervical spine without and with IV contrast OR MRI cervical spine without IV contrast	19 (4.3%)	MRI cervical spine without IV contrast	201 (48.9%)	MRI cervical spine	277 (62.5%)
MRI cervical spine without IV contrast	270 (60.5%)	CT cervical spine without IV contrast	49 (11.9%)	CT cervical spine	80 (18.1%)
CT cervical spine without IV contrast	63 (14.1%)	Nil	161 (39.2%)	Nil	86 (19.4%)
Nil	94 (21.1%)				
**Imaging recommendations for the best imaging tests for replacing CT Cervical Spine**					
**ACR AC**	***n*** **(*n*/153, %)**	**ESR iGuide**	***n*** **(*n*/153, %)**	**RCR iRefer**	***n*** **(*n*/153, %)**
Nil	153 (100%)	MRI Cervical Spine without IV Contrast	1 (0.7%)	MRI Cervical Spine	3 (2.0%)
		Nil	152 (99.3%)	Nil	150 (98%)

## Discussion

This study demonstrated a minimal difference in the proportion of positive and negative radiological findings across the recommendations from the three guidelines, with larger differences observed in procedures classified as appropriate. While the findings may be deemed clinically negligible, in general, we observed variability in the positivity rate across all the imaging recommendations, with appropriate imaging accounting for most of the positive findings.

In our study, CT cervical spine detected a larger number of positive findings, there were findings (*n* = 3/10; 30%) that were categorised as not clinically significant (injuries that, if not identified, would be highly unlikely to result in any harm to patients and require no specific intervention) according to the National Emergency X-Radiography Utilisation Study (NEXUS) [11]. Indeed, for these patients, none required immediate intervention; they were either admitted to the observation ward or to other medical disciplines, or they were referred to a spine surgeon as an outpatient. In other words, the incidence of cervical spine injury in our study was 6.5% (*n* = 10/153), reduced to 4.6% (*n* = 7) after taking into account injuries that were clinically not significant. Our results mirrored those of another study that also showed a decrease in the incidence rate after accounting for clinically significant injury [13].

As imaging referral decisions are currently based on the physician’s acumen, when we compared the requested/performed procedure (X-ray/CT) and the clinical indication(s) to the imaging referral guidelines, a significant number of patients did not receive the recommended best test. Most of the guidelines recommended MRI as the best test to address the clinical scenario, with CT being the best test to replace X-ray for certain clinical scenarios. For example, according to ACR AC, X-ray cervical spine for patients who presented with chronic cervical pain with radiculopathy is categorised as “*May Be Appropriate (Disagreement)*”, while an MRI Cervical Spine is classified as “*Usually Appropriate*”. Likewise, for trauma patients with no unstable injury but kept in a collar for neck pain, CT cervical spine without IV contrast would be “Usually Appropriate” rather than an X-ray, which would be “*May Be Appropriate (Disagreement)*”. This highlights an opportunity to optimise imaging in the ED, particularly when justification and limiting the use of low-dose diagnostic ionising radiation to specific clinical indications are paramount [14, 15].

Indeed, MRI is not currently available to our local ED physicians for imaging referral. While MRI is often considered the best test in many clinical scenarios, it may not significantly improve patient management. It would be ineffective for our large cohort of patients, many of whom (~ 50%) presented with cervical radiculopathy, for cervical spine X-ray, because there are high rates of both false-negative and false-positive results when compared to the gold standard of clinical assessment by specialists [16]. A study found that although there was a significant increase in emergency MRI use following the installation of an easily accessible MRI unit in the ED, however, patients undergoing MRI had increased ED length of stay but decreased admission rates, and associated hospital length of stay post-admission decreased [17]. Indeed, some patients locally had to be admitted to have an MRI investigation performed, which contributed to the admission rates and potentially overloading the inpatient MRI services, which resulted in increased hospital length of stay due to the prolonged wait time for MRI investigations. Furthermore, studies conducted on neuro-related conditions suggested lower long-term direct costs and higher cumulative quality-adjusted life years [18, 19]. Therefore, an opportunity exists to improve work procedures related to imaging referrals in the ED, empowering ED physicians to request imaging that has been “pre-authorised” according to the guidelines’ imaging recommendations. This will eliminate the need for physicians to seek radiologists’ approval for imaging referrals, frequently a source of frustration in a busy ED environment [20]. Consequently, encourages the uptake of imaging referral guidelines in clinical practice.

Despite imaging referral guidelines and associated cervical spine rules/criteria being around for years with a strong evidence base, their uptake has varied. This may be due to detractors such as the physicians’ awareness, acceptance, and concern about applicability to local contexts and the “age” of certain cervical spine rules and criteria (NEXUS and Canadian C-Spine Rules, first described in 1992 and 2001, respectively) [21]. Moreover, the imaging practices of physicians may be driven by habit or anecdote and therefore require a change of mindset among them [22]. Notably, the guidelines indicated imaging in all instances of fractures in traumatic patients. Therefore, our findings provide additional evidence and advocacy for optimisation and appropriate imaging in the ED, as imaging referral guidelines continue to be effective in excluding a positive finding in traumatic and non-traumatic patients, especially when imaging is not indicated.

While our findings supported the aforementioned, the specific recommendations from the guidelines varied, particularly in RCR iRefer. All instances of fractures in the X-ray and CT cohort had “*Usually Appropriate*” recommendations by ACR AC and ESR iGuide, whereas RCR iRefer had a recommendation of “*Indicated only in specific circumstances*”. RCR iRefer has classified these imaging procedures as non-routine investigations, which are usually only undertaken if a clinician provides cogent reasons or if they are justified by a radiologist. Despite all guidelines demonstrating adequacy in this sample, ACR AC and ESR iGuide clearly recommended and rated the imaging as indicated, a recommendation that RCR iRefer agreed with, albeit with a lower “rating”. Indeed, RCR iRefer classified these imaging investigations as appropriate rather than most likely to contribute to the clinical diagnosis and management. The situation may present some challenges to end-users of the guideline, particularly the junior clinicians who have less clinical experience. In our local ED, most imaging, particularly X-rays, is requested by junior clinicians who prefer directive recommendations [23]. To better meet the needs of this group of end-users, the ACR AC and ESR iGuide are better suited than the supportive recommendations from RCR iRefer, which are more appropriate for senior clinicians with substantial clinical experience.

Though our findings may be clinically insignificant, they may be a result of the study being conducted in the ED of an academic hospital, where there may be a lower baseline percentage of inappropriate imaging referrals, leaving little room to demonstrate the true extent of the difference in the proportion of positive and negative radiological findings [24]. We also have to acknowledge that there are situations where clinical judgement and local practice might necessitate deviation from imaging recommendations. Such situations include localised trauma protocols (definition of a high-speed motor vehicle collision, etc.) and patient age groups, which commonly influence the decision to proceed with imaging. Furthermore, although our study did not include paediatric patients, it is important to note that this distinct population group may require deviations from the recommended protocols or guidelines, as clinical judgement often takes precedence.

Nonetheless, our approach of longitudinally monitoring patients until their disposition allowed us to elucidate physicians’ risk for patient safety and quality of care, in particular addressing the question of “What impact will there be on patient outcome if I follow/do not follow imaging recommendations from the guidelines?” Through this study, we have defined best practices for using imaging referral guidelines in a clinical setting and demonstrated the impact of the intervention on patient safety, outcomes, and risk. We believe that addressing the physicians’ risk will facilitate increased guideline trust among physicians and encourage review of current practice and, consequently, a steady march towards practice and mindset change.

### Limitations

To our knowledge, this is the first study exploring the imaging characteristics and patient disposition among the imaging recommendations from the three prominent radiology professional bodies: ACR, ESR, and RCR. However, our study has several limitations. One limitation is the retrospective nature of our study, whereby the available data is restricted to information that was documented in the electronic medical records by ED physicians at the time of the patient’s visit, specifically the quality of the documentation, which may affect the categorisation of imaging recommendations [25].

Another limitation was the potential for reviewer bias due to the nonblinded review of ED clinical notes; however, we are confident that the objective nature of the collected information and the blinded review of the notes by three reviewers ensured reviewer bias was minimised. Additionally, a difference in the granularity of the guidelines label may affect the classification of the imaging recommendations because we did not consider any descriptor beyond the label of the guidelines (i.e., unlike ACR AC and ESR iGuide, which have descriptors in the guidelines label, RCR iRefer has a description of the specific circumstance in which to consider the test separated from the guideline’s label). Furthermore, as it is beyond the scope of the study to perform longitudinal follow-up on the individual patient, we do not have information on the subsequent management or imaging of these patients, who may be discharged, referred to a specialist, or admitted. Therefore, it is important to interpret our positivity and negativity rates with caution.

## Conclusion

There were differences in the rates of positive significant findings within all the imaging recommendations for cervical spine, albeit minimal. Although the differences may be clinically negligible, when imaging is considered inappropriate, imaging referral guidelines can effectively exclude clinically significant injury due to their high specificity. Furthermore, the analysis of patient dispositions suggests that we can omit most of this inappropriate imaging and still maintain safety and quality of care in the ED. It is therefore vital for imaging referral guidelines to be integrated into clinical practice in the ED, given that inappropriate imaging is preventable.

## Supplementary information

ELECTRONIC SUPPLEMENTARY MATERIAL

## Data Availability

The data that supports the findings of this study are available from the corresponding author upon reasonable request.

## Abbreviations

ACR AC: American College of Radiology Appropriateness Criteria
CIRB: Centralised Institutional Review Board
ED: Emergency Department
ESR: European Society of Radiology
NEXUS: National Emergency X-Radiography Utilisation Study
RCR: Royal College of Radiologists
