# Supplementary material for: Positivity rates and subsequent patient dispositions after utilisation of cervical spine imaging referral guidelines in Singapore
Source: Insights Imaging. 2025 Aug 8;16:170. doi: 10.1186/s13244-025-02048-9 (PMC12334777; doi:10.1186/s13244-025-02048-9)
Supplement: Supplementary file 1 — ELECTRONIC SUPPLEMENTARY MATERIAL [file 13244_2025_2048_MOESM1_ESM.pdf]

Positivity rates and subsequent patient dispositions after utilisation of cervical spine imaging referral guidelines in Singapore

ELECTRONIC SUPPLEMENTARY MATERIAL

**Supplemental Material Table 1: Evaluation of imaging referrals' indications based on imaging referral guidelines**	
**X-ray Cervical Spine imaging referrals**	
**ACR Appropriateness Criteria**	**n *(n/452, %)***
Acute onset myelopathy. Initial imaging.	6 (1.3%)
Age greater than or equal to 16 years and less than 65 years. Suspected acute blunt cervical spine trauma; imaging not indicated by NEXUS or CCR clinical criteria. Patient meets low risk criteria. Initial imaging.	83 (18.4%)
Age greater than or equal to 16 years. Suspected acute cervical spine blunt trauma. Obtunded patient with no traumatic injury identified on cervical spine CT. Next imaging study after CT cervical spine without IV contrast.	1 (0.2%)
Age greater than or equal to 16 years. Suspected acute cervical spine blunt trauma. Clinical or imaging findings suggest ligamentous injury. Next imaging study after CT cervical spine without IV contrast.	1 (0.2%)
Age greater than or equal to 16 years. Suspected acute cervical spine blunt trauma. Imaging indicated by NEXUS or CCR clinical criteria. Initial imaging.	56 (12.4%)
Cervicogenic headache and new or increasing nontraumatic cervical or neck pain. No neurologic deficit. Initial imaging.	28 (6.2%)
Chronic cervical or neck pain. Initial imaging.	11 (2.4%)
Chronic or progressive myelopathy. Initial imaging.	7 (1.5%)
Known malignancy. New or increasing nontraumatic cervical or neck pain or radiculopathy. Initial imaging.	6 (1.3%)
New or increasing nontraumatic cervical or neck pain. No "red flags." Initial imaging.	239 (52.9%)
Prior cervical spine surgery. New or increasing nontraumatic cervical or neck pain or radiculopathy. Initial imaging.	7 (1.5%)
Suspicion for infection with new or increasing nontraumatic cervical or neck pain or radiculopathy. Initial imaging.	1 (0.2%)
Does not align with any criteria	6 (1.3%)
**ESR iGuide**	**n *(n/452, %)***
Neck trauma - uncomplicated (NEXUS/CCR neg)	91 (20.1%)
Neck trauma, dangerous injury mechanism	39 (8.6%)
Neck trauma, focal neuro deficit or paresthesia	2 (0.4%)
Neck trauma, impaired ROM	3 (0.7%)
Neck trauma, intoxicated or obtunded	1 (0.2%)
Neck trauma, midline tenderness	4 (0.9%)

Radiculopathy	201 (44.5%)
Neck pain, initial exam	56 (12.4%)
Bone neoplasm suspected, C-spine, initial exam	1 (0.2%)
Neck Pain, chronic, history of malignancy, initial exam	6 (1.3%)
Neck pain, chronic, prior spine surgery, initial exam	7 (1.5%)
Does not align with any criteria	41 (9.1%)
**RCR iRefer**	**n (n/452, %)**
Cervical spine assessment in conscious patient with head and/or facial injury only	59 (13.1%)
Headache	28 (6.2%)
Neck injury with pain	80 (17.7%)
Non-traumatic neck pain, brachialgia, degenerative change	277 (61.3%)
Does not align with any criteria	8 (1.8%)
**CT Cervical Spine imaging referrals**	
**ACR Appropriateness Criteria**	**n *(n/153, %)***
Age greater than or equal to 16 years and less than 65 years. Suspected acute blunt cervical spine trauma; imaging not indicated by NEXUS or CCR clinical criteria. Patient meets low risk criteria. Initial imaging.	14 (9.2%)
Age greater than or equal to 16 years. Suspected acute cervical spine blunt trauma. Imaging indicated by NEXUS or CCR clinical criteria. Initial imaging.	137 (89.5%)
Age greater than or equal to 16 years. Acute cervical spine injury detected on radiographs. Treatment planning for mechanically unstable spine.	2 (1.3%)
**ESR iGuide**	**n *(n/153, %)***
Neck trauma - uncomplicated (NEXUS/CCR neg)	14 (9.2%)
Neck trauma, dangerous injury mechanism	75 (49.0%)
Neck trauma, focal neuro deficit or paresthesia	15 (9.8%)
Neck trauma, impaired ROM	2 (1.3%)
Neck trauma, intoxicated or obtunded	26 (17.0%)
Neck trauma, midline tenderness	19 (12.4%)
Neck trauma, myelopathy	1 (0.7%)
C-spine fracture, traumatic	1 (0.7%)
**RCR iRefer**	**n *(n/153, %)***
Cervical spine assessment in conscious patient with head and/or facial injury only	131 (85.6%)
Neck injury with pain	17 (11.1%)
Cervical spine assessment in unconscious patient with head injury	2 (1.3%)
Neck injury with neurological deficit	3 (2.0%)

**Supplemental Material Table 2: Imaging characteristics for X-ray and CT Cervical Spine**			
**X-ray**	**n (n/452, %)**	**CT**	**n (n/153, %)**
Positive	5 (1.1%)	Positive	10 (6.5%)
Negative	447 (98.9%)	Negative	143 (93.5%)
**X-ray Findings**		**CT Findings**	
Fracture*	2 (0.4%)	Fracture*	10 (6.5%)
Prevetebral soft tissue swelling*	3 (0.7%)	Cervical Spondylosis	113 (73.9%)
Cervical Spondylosis	382 (84.5%)	Spondylolisthesis	40 (26.1%)
Spondylolisthesis	144 (31.9%)	Ossification of the Posterior Longitudinal Ligament	19 (12.4%)
Ossification of the Posterior Longitudinal Ligament	3 (0.7%)	Ossification of the Anterior Longitudinal Ligament	3 (2.0%)
Loss of the normal cervical lordosis	150 (33.2%)	Diffuse idiopathic skeletal hyperostosis	2 (1.3%)
Diffuse idiopathic skeletal hyperostosis	3 (0.7%)	Calcium pyrophosphate dihydrate	4 (2.6%)
		Longus Colli Calcific Tendinitis	1 (0.7%)
		Ossification ligamentum flavum	1 (0.7%)
*Radiological findings classified as positive, significant findings in the study			

**Positive findings, associated patient disposition and imaging recommendation**					
**X-ray**					
**No.**	**Positive Significant Finding(s)**	**Disposition**	**ACR AC**	**ESR iGuide**	**RCR iRefer**
1	There is prevertebral soft tissue swelling noted anterior to C2 to C4 vertebrae, measuring up to 0.9 cm. The aetiology of this prevertebral soft tissue swelling is uncertain.	Admitted to Other Discipline	May Be Appropriate (Disagreement)	May Be Appropriate	Indicated only in specific circumstances
2	Linear lucency across the C5 vertebral body is suspicious for a fracture. Ovoid lucency	Admitted to Other Discipline	Usually Appropriate	Usually Appropriate	Indicated only in specific circumstances

	in the anteriosupoerior aspect of the C5 may be related to the fracture.				
3	There appears to be prevertebral soft tissue swelling at the C6-T1 levels.	Discharged<br><br>(At Own Risk)	May Be Appropriate	May Be Appropriate	Indicated only in specific circumstances
4	There is apparent mild thickening of prevertebral soft tissues, which is indeterminate for postsurgical change or development of paraspinal/retropharyngeal collection.	Referred to Spine Surgeon (Outpatient)	Usually Appropriate	Usually Appropriate	Indicated only in specific circumstances
5	There is flattening of C4-C6 suggestive of compression fractures.	Referred to Spine Surgeon (Outpatient)	Usually Appropriate	Usually Appropriate	Indicated only in specific circumstances
**CT**					
**No.**	**Positive Significant Finding(s)**	**Disposition**	**ACR AC**	**ESR iGuide**	**RCR iRefer**
1	Hyperdensities in the epidural region with mass effect upon the cervical cord, worrying for epidural hematoma. Acute non-displaced fracture of the left transverse process of C6.	Admitted to Orthopaedic Surgery	Usually Appropriate	Usually Appropriate	Indicated only in specific circumstances
2	Acute fractures of the left sided posterior elements from C4 to C6, with extension to the C5 left transverse foramen.<br><br>Right C7 transverse process fracture.<br><br>Suggestion of an undisplaced fracture at the C6 anterior inferior	Admitted to Orthopaedic Surgery	Usually Appropriate	Usually Appropriate	Indicated only in specific circumstances

	corner left lateral aspect.				
3	Acute minimally displaced fractures are detected at C1 right anterior arch and right lateral mass, as well as the C2 right transverse process.	Admitted to Orthopaedic Surgery	Usually Appropriate	Usually Appropriate	Indicated only in specific circumstances
4#	Moderate wedge compression fracture of the T2 vertebral body.	Admitted to Observation Ward	Usually Appropriate	Usually Appropriate	Indicated only in specific circumstances
5	Fracture of the C5 spinous process extending to the right lamina. Minimally displaced fractures of the right C5 and left C6 transverse processes involving the bilateral transverse foramina.	Admitted to Orthopaedic Surgery	Usually Appropriate	Usually Appropriate	Indicated only in specific circumstances
6#	Undisplaced fractures of the C4 and C5 left transverse processes. Possible disruption of the left C4 and C5 transverse foramina.	Admitted to Other Discipline (Acute Care Surgery)	Usually Appropriate	Usually Appropriate	Indicated only in specific circumstances
7	Fracture of the right inferior articular process of C4. It is uncertain whether this represents an acute or chronic injury.	Referred to Spine Surgeon (Outpatient)	Usually Appropriate	Usually Appropriate	Indicated only in specific circumstances
8	Teardrop fracture at the anteroinferior aspect of the C5 vertebral body.<br><br>Fracture of the left C4 superior articular process.	Admitted to Orthopaedic Surgery	Usually Appropriate	Usually Appropriate	Indicated only in specific circumstances
9#	Right C7 transverse process linear lucency, visualised only on the	Admitted to Other Discipline	Usually Appropriate	Usually Appropriate	Indicated only in specific circumstances

	sagittal sequence, is equivocal for an undisplaced fracture.				
10	Irregular linear lucency seen traversing the C7 vertebral body, raises suspicion for an undisplaced C7 vertebral body fracture. Equivocal density along the posterior epidural space at C2 to C7, indeterminate for ligamentum flavum hypertrophy vs small epidural haematoma.	Admitted to Orthopaedic Surgery	Usually Appropriate	Usually Appropriate	Indicated only in specific circumstances

Supplemental Material Table 3: Patient Disposition after Imaging		
**X-ray**		
**ACR AC**	**Disposition**	
Usually Appropriate	Treated and discharged	n=18
	Referred to Spine Surgeon (Outpatient Appointment)	n=20
	Referred to General Orthopaedic (Outpatient Appointment)	n=3
	Referred to Other Discipline (Outpatient Appointment)	n=2
	Admitted to Orthopaedic Surgery	n=2
	Admitted to Other Discipline	n=18
	Admitted to Observation Ward (in ED)	n=1
May Be Appropriate	Treated and discharged	n=13
	Referred to Spine Surgeon (Outpatient Appointment)	n=11
	At Own Risk	n=1
	Referred to General Orthopaedic (Outpatient Appointment)	n=2
	Referred to Other Discipline (Outpatient Appointment)	n=16
	Admitted to Orthopaedic Surgery	n=4
	Admitted to Other Discipline	n=36
	Admitted to Observation Ward (in ED)	n=5
May Be Appropriate (Disagreement)	Treated and discharged	n=35
	Referred to Spine Surgeon (Outpatient Appointment)	n=65
	At Own Risk	n=2
	Referred to General Orthopaedic (Outpatient Appointment)	n=12
	Referred to Other Discipline (Outpatient Appointment)	n=9
	Admitted to Orthopaedic Surgery	n=6
	Admitted to Other Discipline	n=65
Usually Not Appropriate	Treated and discharged	n=39
	Referred to Spine Surgeon (Outpatient Appointment)	n=22
	Referred to General Orthopaedic (Outpatient Appointment)	n=11
	Referred to Other Discipline (Outpatient Appointment)	n=7
	Admitted to Orthopaedic Surgery	n=6

	Admitted to Other Discipline<br>Admitted to Observation Ward (in ED)	n=11<br>n=4
**ESR iGuide**	**Disposition**	
Usually Appropriate	Treated and discharged<br>Referred to Spine Surgeon (Outpatient Appointment)<br>Referred to General Orthopaedic (Outpatient Appointment)<br>Referred to Other Discipline (Outpatient Appointment)<br>Admitted to Orthopaedic Surgery<br>Admitted to Other Discipline<br>Admitted to Observation Ward (in ED)	n=16<br>n=19<br>n=3<br>n=2<br>n=4<br>n=25<br>n=1
May Be Appropriate	Treated and discharged<br>Referred to Spine Surgeon (Outpatient Appointment)<br>At Own Risk<br>Referred to General Orthopaedic (Outpatient Appointment)<br>Referred to Other Discipline (Outpatient Appointment)<br>Admitted to Orthopaedic Surgery<br>Admitted to Other Discipline<br>Admitted to Observation Ward (in ED)	n=45<br>n=72<br>n=3<br>n=13<br>n=14<br>n=9<br>n=89<br>n=5
Usually Not Appropriate	Treated and discharged<br>Referred to Spine Surgeon (Outpatient Appointment)<br>Referred to General Orthopaedic (Outpatient Appointment)<br>Referred to Other Discipline (Outpatient Appointment)<br>Admitted to Orthopaedic Surgery<br>Admitted to Other Discipline<br>Admitted to Observation Ward (in ED)	n=42<br>n=19<br>n=11<br>n=7<br>n=2<br>n=5<br>n=4
**RCR iRefer**	**Disposition**	
Indicated only in specific circumstances	Treated and discharged<br>Referred to Spine Surgeon (Outpatient Appointment)<br>At Own Risk<br>Referred to General Orthopaedic (Outpatient Appointment)	n=103<br>n=118<br>n=3<br>n=28

	Referred to Other Discipline (Outpatient Appointment)<br>Admitted to Orthopaedic Surgery<br>Admitted to Other Discipline<br>Admitted to Observation Ward (in ED)	n=33<br>n=18<br>n=130<br>n=10
**CT**		
**ACR AC**	**Disposition**	
Usually Appropriate	Treated and discharged<br>Referred to Spine Surgeon (Outpatient Appointment)<br>At Own Risk<br>Referred to General Orthopaedic (Outpatient Appointment)<br>Referred to Other Discipline (Outpatient Appointment)<br>Admitted to Orthopaedic Surgery<br>Admitted to Other Discipline<br>Admitted to Observation Ward (in ED)	n=9<br>n=7<br>n=2<br>n=1<br>n=1<br>n=39<br>n=75<br>n=5
Usually Not Appropriate	Referred to General Orthopaedic (Outpatient Appointment)<br>Admitted to Orthopaedic Surgery<br>Admitted to Other Discipline<br>Admitted to Observation Ward (in ED)	n=1<br>n=4<br>n=7<br>n=1
**ESR iGuide**	**Disposition**	
Usually Appropriate	Treated and discharged<br>Referred to Spine Surgeon (Outpatient Appointment)<br>At Own Risk<br>Referred to General Orthopaedic (Outpatient Appointment)<br>Referred to Other Discipline (Outpatient Appointment)<br>Admitted to Orthopaedic Surgery<br>Admitted to Other Discipline<br>Admitted to Observation Ward (in ED)	n=9<br>n=7<br>n=2<br>n=1<br>n=1<br>n=39<br>n=75<br>n=5
Usually Not Appropriate	Referred to General Orthopaedic (Outpatient Appointment)<br>Admitted to Orthopaedic Surgery<br>Admitted to Other Discipline	n=1<br>n=4<br>n=7

	Admitted to Observation Ward (in ED)	n=1
**RCR iRefer**	**Disposition**	
Indicated	Referred to General Orthopaedic (Outpatient Appointment)	n=1
	Admitted to Orthopaedic Surgery	n=5
	Admitted to Other Discipline	n=5
Indicated only in specific circumstances	Treated and discharged	n=9
	Referred to Spine Surgeon (Outpatient Appointment)	n=7
	At Own Risk	n=2
	Referred to General Orthopaedic (Outpatient Appointment)	n=1
	Referred to Other Discipline (Outpatient Appointment)	n=1
	Admitted to Orthopaedic Surgery	n=38
	Admitted to Other Discipline	n=78
	Admitted to Observation Ward (in ED)	n=6
